# Debt, shame, and survival: becoming and living as widows in rural Kerala, India

**DOI:** 10.1186/1472-698X-12-28

**Published:** 2012-11-06

**Authors:** Katia Sarla Mohindra, Slim Haddad, Delampady Narayana

**Affiliations:** 1 Institute of Population Health, University of Ottawa, Ottawa, Canada; 2 Centre de recherche du Centre Hospitalier de l’Université de Montréal, Montréal, Canada; 3 Gulati Institute of Finance and Taxation, Thiruvananthapuram, India

## Abstract

**Background:**

The health and well-being of widows in India is an important but neglected issue of public health and women’s rights. We investigate the lives of Indian women *as they become widows*, focussing on the causes of their husband’s mortality and the ensuing consequences of these causes on their own lives and identify the opportunities and challenges that widows face in living healthy and fulfilling lives.

**Methods:**

Data were collected in a Gram Panchayat (lowest level territorial decentralised unit) in the south Indian state of Kerala. Interviews were undertaken with key informants in order to gain an understanding of local constructions of ‘widowhood’ and the welfare and social opportunities for widows. Then we conducted semi-structured interviews with widows in the community on issues related to health and vulnerability, enabling us to hear perspectives from widows. Data were analysed for thematic content and emerging patterns. We synthesized our findings with theoretical understandings of vulnerability and Amartya Sen’s entitlements theory to develop a conceptual framework.

**Results:**

Two salient findings of the study are: first, becoming a widow can be viewed as a type of ‘shock’ that operates similarly to other ‘economic shocks’ or ‘health shocks’ in poor countries except that the burden falls disproportionately on women. Second, widowhood is not a static phenomenon, but rather can be viewed as a multi-phased process with different public health implications at each stage.

**Conclusion:**

More research on widows in India and other countries will help to both elucidate the challenges faced by widows and encourage potential solutions. The framework developed in this paper could be used to guide future research on widows.

## Background

The health and well-being of widows in India is an important but neglected issue of public health and women’s rights. According to the 2001 Census, widows represent 9% of the female population (over 34 million women), yet researchers and policy makers have paid scant attention to this group. This may partly be due to the overemphasis of the ‘instrumental’ (as opposed to intrinsic) value of women in society, the religious symbolism of widowhood in India, and the view that widows are a private matter rather than a social problem. [1-4] There is a need for a better understanding of widows beyond the sensational cases such as *sati* (widow burning), to include the more subtle, yet widespread deprivations [1].

Widows have been identified as a vulnerable group in India, but what does this vulnerability entail? Simply becoming a widow lowers a woman’s social status, as she succumbs to a ‘social death’ when her husband dies – she may even be viewed as inauspicious, especially if she is too young or lost her husband soon after marriage. Widows also face challenges stemming from both a set of social restrictions that have been placed on them and a lack of social protection. These issues have been well documented and include the following: social isolation due to their obligation to remain in their husband’s village, restrictions in employment opportunities, legal difficulties in defending their rights over husband’s property, inadequate support from their husband’s family, and limited economic support in the absence of an adult son. [1,5,6] Relatively less is known about the circumstances and implications of the death of a woman’s husband, as well as the health and welfare outcomes of widows. There is some evidence stemming from econometric and demographic studies undertaken during the 1990s suggesting that widows face an increased mortality and higher levels of impoverishment. [7,8] Ethnographic studies have further demonstrated high levels of impoverishment and social suffering faced by widows [1].

This paper presents findings from a qualitative study undertaken in a community in the South Indian state of Kerala in order to contribute to a more comprehensive understanding of the vulnerability of widows. The aims of this study were two-fold. First, to examine the lives of women *as they become widows*, focussing on the causes of their husband’s mortality and the ensuing consequences of these causes on their own lives. Second, to identify the opportunities and challenges that widows face in living healthy and fulfilling lives. Based on the study findings, we develop a conceptual framework that could be used to guide future research on widows in India (and in other countries with similar levels of discrimination) and to help guide interventions to improve the health and well-being of widows.

### Widows in Kerala

There is a slightly higher proportion of widows in Kerala (10%) compared to the Indian average. This may be attributed to Kerala’s demographic profile (i.e. higher female life expectancy), a greater age differential at marriage, and low remarriage rates among widows [9]. The studies on the welfare of widows have focussed on widowhood in northern India, where they face greater levels of discrimination compared to their southern counterparts in general and Kerala in particular. Women in Kerala are known for their elevated status. The gender bias in sex ratios found in India is absent in Kerala (the female to male ratio is 1,058 and 933 per 1,000 for Kerala and India respectively). [10] While life expectancy for Indian women and men is almost identical (61 years for women and 60 years for men), Kerala reports female advantage (76 years for women and 70 years for men) and the highest female life expectancy in the country.

The gender gap in literacy rates is relatively narrow (88% for women and 94% for men in 2011), fertility rates are low (below replacement levels). It has been argued, however, that these indicators only tell part of the story; patriarchal norms continue to reign over the lives of women, manifesting in various forms, including domestic violence. [11,12] It is also reported that Kerala has the worst record among the Indian states for economic dependency among the elderly; 76% of its elderly women reporting no financial asset in their name. [13] While, there is a history of progressive public policies in this state, including public support for vulnerable groups, such as widows, [6] economic opportunities for women are few raising specific concerns for their maintaining a good standard of living—particularly if the husband has passed away prematurely. In this case, a woman may be left with young children and no source of livelihood. Whereas in the case of a husband’s death after leading a full life and having grown up children who are shouldering family responsibilities, the impact on a woman’s life would likely be much less, particularly if an adequate social security system is in place.

### Study setting

The study was conducted in Kottathara, a *gram panchayat* (the lowest territorial decentralized unit) in the rural district of Wayanad, locale of an action research project, implemented by the Centre for Development Studies and the *Université de Montréal*. The project obtained ethical approval by the *Université de Montréal* Ethics Committee on April 25, 2003. One of the main goals of the project was to develop an evidence base that will contribute to the reduction of inequalities in health care and other basic services. The project has several databases, including a baseline survey (a census conducted in 2003 of the 3,352 households located in the *gram panchayat*, which included information on demographics and socio-economic conditions of the population).

The study site is multi-religious and multi-caste (caste is a hereditary, endogamous, usually localised group, having a traditional association with an occupation and a particular position in the hierarchy of castes; each caste is ranked in a hierarchical order), with a high concentration of indigenous populations, known as Scheduled Tribes (31%). Social inequalities in women’s health have been observed across socioeconomic and caste groups. [14] Financial barriers to accessing quality health care have been identified, particularly among the poorest and marginalised groups. [15] There are high levels of unemployment and limited economic opportunities. Most of the population are engaged in agriculture and the majority of workers are cultivators or wage workers. Recent declines in agricultural prices in the state have negatively impacted the economy. For example, the price of pepper, one of the main crops grown in the region, dropped from Rs. 20, 506 per 100 kg in 1999/2000 to Rs. 7, 814 per 100 kg in 2002/03, [16,17] now gone up to 5000 (The Indian currency is known as rupees, or Rs., when citing actual costs; 1 USD is approximately 46 rupees, currently 55 rupees). A recent drought had exacerbated the situation and there have been increasing stories of mounting debts of farmers, and incidences of debt-related suicides.

The following information is derived from the baseline survey. There are 759 widows living in Kottathara, 9.3% of all females, compared to only 90 widowers or 1.1% of the male population. The widow to widower ratio is 8.4 and the mean age of widows is 60 years. Significantly, the median age is also 60 years, which is due especially to the large percentage of widows below 60 years among the Scheduled Castes and Scheduled Tribes (53%) and Muslims (42%). Among widows, 68% are the head of the household.

## Methods

### Data collection

Field work was conducted over a period of 2 months in 2004, involving a two step process. First, we interviewed key informants in order to gain an understanding of local constructions of ‘widowhood’ and the welfare and social opportunities for widows. The key informants included: 2 local government employees, 2 members of a local NGO, and 3 widows from a neighbouring *gram panchayat*. Second, we conducted semi-structured interviews with 10 widows in the community on issues related to health and vulnerability, enabling us to hear perspectives from widows. Using the project’s baseline survey, we purposely selected widows that would sample a variety of characteristics, including religion, caste or tribe affiliations, age, and poverty status of the household. We interviewed women ranging in age from 33 to 73 years with diverse religious and caste affiliations, including Muslims, Christians, Hindu women from both forward and low caste groups, and two women were from the Paniya tribe, a marginalised Scheduled Tribe. Six women came from households that were below the poverty line.

The median duration of widowhood was 15 years (Table 1). All the women surveyed have children, most women have between three and four children (boys and girls); only one participant did not have a son. Most of the participants now live with their adult children and were considered to be the head of the household (Table 1). Seven women have access to land, three of whom have landholdings in their own name (Table 1).

**Table 1:** **Age when widowed**, **household and living arrangements**, **and land ownership**

**Pseudonym**	**Age when widowed**	**Head of household**	**Living arrangements**	**Landholdings**
Geeta	27	No	Lives with parents	Not in possession of her husband’s land
Elsamma	27	Yes	Lives alone	1 acre divided into 8 equal shares for her children and herself (from husband)
Amina	37	Yes	Lives with sons	19 cents of land divided for children and herself, 5 cents in her name (from husband)
Jamila	63	Yes	Lives with eldest son	7 cents of land in her name (from husband)
Lata	35	Yes	Lives with children in tribal colony	Colony has 10 cents of land (from Panchayat)
Priya	30	Yes	Lives with son	73 cents of land in her name (from husband)
Usha	31	Yes	Lives with daughters	35 cents (5 cents from husband, 30 cents from father)
Sumathi	57	Yes	Lives with daughters	-
Leela	21	Yes	Lives with parents	4 cents of land (from Panchayat)
Shantha	46	Yes	Lives with eldest daughter	

Source: Qualitative survey.

The interview guide consisted of open-ended questions, which was designed in conjunction with the director of a local non governmental organization (NGO), who has more than 20 years experience working with women and development projects in the area (see Additional file 1). We included a limited number of simple questions in order to ease translation and that were to help prompt more in-depth discussions by encouraging the participants to share their experiences and stories. The guide was pre-tested and revised to ensure that the questions were clear and appropriate. Questions were asked regarding the circumstances of becoming widows, survival strategies used following the death of a husband, health and well-being, and the nature and level of support received from their families, the government, and the community (See Annex for interview guide). Interviews were conducted until the point of saturation (N=10), which although it is a relatively small sample, represented 13% of the widows living in the study site. Three women identified for interviews were unable to participate because they were not presently residing in their household.

The interviews were conducted by a local team (two females from a local NGO were trained as interviewers and note takers) under the supervision of the first author, a woman of North Indian descent. Informed signed consent was obtained prior to interviews. The interviews were performed in the local language of *Malayalam* and detailed notes were taken. The first author also participated in the interviews by asking follow-up questions after the interview was completed, with the aid of a local translator. Interviews were conducted at the homes of the participants and whenever possible the interviews were conducted in a private area in order to allow the widow to speak freely, without the presence of family members. Interviews lasted between 30 and 45 min. The interview notes were translated from *Malayalam* to English by a local translator and the transcripts were reread for logical translation by the research team.

### Analysis

Data analysis proceeded concurrently with data collection. Debriefing sessions were held following each interview with the field team and the NGO director. Line by line coding of transcripts and field notes were undertaken. Data were analysed for thematic content and emerging patterns. The data was grouped into themes and sub-themes and the interrelations between these sub-categories. A report of the preliminary findings was prepared and circulated among the project team for further discussion. Pseudonyms have been used for the participants of this study in order to protect anonymity. We have also changed the names of the people the women talked about in their interviews (e.g. other family members, physicians) as well as the names of institutions and some of the locations mentioned in the interviews.

## Results

In response to the first study question (becoming a widow), three themes emerged: debt, shame, and survival. In addition, we identified two distinct ‘profiles of widows’, based on the cause of death of their husband. Three themes emerged in response to the second study question (opportunities and challenges to leading healthy and fulfilling lives), economic security, social mobility and social exclusion, and access to health care. The results are organised under these themes, with illustrative examples.

### Becoming widows: debt, shame, and survival

During the initial period of widowhood, women faced issues related to debt, shame, and survival. Following a mourning period (which varies across castes and religious groups), the women expressed a sense of hopelessness, related both to the experience and emotional stress of losing their husbands and the challenges in meeting the basic needs of their households. Nine of the widows interviewed had lost their husband at a time when their children were still young. None of them reported receiving substantial support from their in-laws, although the women did speak of receiving at least some support from the community. The women remained in their own households after becoming widows, with the exception of two who had returned to live with their parents (Table 1). They became the primary breadwinners of the family, assuming responsibilities of household decision-making. This early period was highly stressful in their lives. In the most extreme case, Elsamma, a Christian woman with forward caste affiliations with 5 children talked about contemplating suicide:

“*Following my husband*’*s death*, *all my children were below 12 years*, *and I had decided to jump in the river and commit suicide*. *I had attempted suicide in this way three times*.”

The widows reported having to cope with the specific circumstances surrounding the deaths of their husbands, both prior to and immediately after their husband’s death. Two main profiles emerged, women who lost their husband due to a chronic illness and women whose husbands had alcohol-related problems and ‘drank themselves to death’. These profiles are described in further detail below.

### Widow profile 1: The chronically ill husband (debt)

Seven widows reported that their husbands had died from a chronic illness, who had been ill for a lengthy period of time before passing away. During this time, the men were often bedridden and had to be hospitalized, at least periodically creating economic and emotional burdens. The women continued to care for young children, met household needs, while struggling to repay debts incurred by the medical costs of their husband’s illness. The case of Amina, a Muslim woman, married at the age of 14 and had four children prior to her husband’s illness, is illustrative:

"“For ten years, my husband had been bed ridden. We spent a lot of money for his health care. During that time I was getting only 20 rupees as a kuli worker [wage labourers working in agricultural sector]. I had to meet all the household expenses. I could send the children to school only up to 4^*th*^standard.” "

In cases where there were insufficient funds, care would be delayed or stopped altogether. This was the case for Sumanthi, a Paniya woman, as she recalled:

"“My husband died six years ago, the cause was not known. He returned home one day complaining of a problem with his head, shortly thereafter he lost his ability to speak. He was taken to Kalipa College. After one month he was shifted back to his home in Kottathara with some medication. He was able to get back some of his ability to speak. The family became unable to pay for the medicine, and it was discontinued. Previously the community had rallied around the family to help pay for the medical fees. He was in this state for two years before he passed away.” "

After becoming widows, the women assumed multiple roles within the household: breadwinner, decision-maker, child raiser, homemaker, and caregivers. Children of widows also had to make sacrifices, helping to take over responsibilities often at the expense of their schooling. And there were still debts to pay: Amina, for example, continued to pay off debts related to her husband’s medical care for another couple of years and her children (who as she described above only attended school up to 4^th^ standard) were never able to return to school.

### Widow profile 2: Husbands who drank themselves to death (shame)

The second profile that emerged was one of women married to men with alcohol-related problems. Alcohol is consumed predominantly by men and it is generally frowned upon by society – although it is readily available. Geeta, Leela and Usha (widowed at 27, 21 and 31 years of age respectively) reported that their husbands had been alcoholics. The use of excessive alcohol was intertwined with shame. This shame was partly felt by the men themselves (in rural Kerala, public drunkenness is viewed negatively by society), as Geeta, a young Hindu woman, recalled:

"“The people were telling me that my husband after having consumed too much alcohol had fallen face down in the vegetable garden. Our neighbour had witnessed the incident and had threatened to report him to the police. It is said that his shame led him to kill himself.” "

Feelings of shame, however, extended to the families in general and the wives in particular. In Geeta’s case, she was blamed by her husband’s family and the community for her husband’s drinking—she was even blamed for his suicide and was often taunted and ridiculed by others. For Leela and Usha their shame was exacerbated by their husband’s abandonment since social shame is associated with abandoned women in India. Their husbands did not assume any financial responsibility for their families and did not maintain any communication with their wives. Leela was abandoned by her husband only to learn from a friend that he had died:

"“My husband was a drunk, who did not look after me. He left me when I was pregnant, and I was 20 years old. I did not know where he had gone. I was told by someone that he had gone for work outside and had been bitten by a snake and died.” "

Usha was abandoned by her husband when she had three young children; “one day he left me to go back to Malagy [a *gram panchayat* in a neighbouring district]”. Usha spoke more passionately and at greater length about her daughter, Beena, who is also a widow and had begun her marriage with great love and happiness:

"“Her husband passed away almost two years ago. Like me, her husband was a drunk. They had a love marriage…Soon after he began keeping bad company and started drinking. He ended up selling the shop and he and Beena moved around Wayanad for work, living in rented houses. He was drinking excessively and was making lots of problems in the home. He was drinking daily and was on occasion beating my daughter and myself. He was good for nothing. The neighbours confronted him and told him to improve his behaviour or leave. He was later hospitalized…where he died of jaundice. She [Beena] received a telegram three days later informing her of her husband’s death…Today Beena is not emotionally well she is depressed…she has no friends and only leaves the house to the angawadi [the preschool where Beena works] and then returns home.”"

### Survival

In both of the profiles of widows, the women spoke about particular hardships during the early periods of their widowhood, having to cope with financial and household matters that had previously been the responsibility of their husband with little to no help. Pata’s statement is representative of most of the women interviewed:

"“I had met with a lot of difficulties at the death of my husband, especially financial difficulties. There was nobody here to help us.” "

The women did feel that they could rely—at least to some extent—on some community support. As Usha put it:

"“The community has always helped whenever there is a problem. There is a mutual understanding among my neighbours to solve problems.” "

However, community support was generally neither sufficient nor sustainable. Priya recalled:

"“I did not receive any support from my husband’s family, but the community helped me in the beginning, although this support has been slowly withdrawn.” "

Therefore; women were faced to take on their families’ survival on their own. A certain level of fear and uncertainty were initially felt. Over time, however, these women assumed their roles in the household with greater confidence and as Priya remarked: “*I feel that my power in the household has been increasing over time*”. Most women expressed that household decision-making became important to do *with* their children, especially as the children got older. Lata, a Paniya woman, stated unequivocally that “*all of my decisions are made in consultation with my children*.” As their children became older and were able to take over some financial responsibilities the women also found life got easier. For Priya, her life got easier when her son married and took on financial responsibilities:

"“I consumed little food at that time, [the early period following the death of Priya’s husband] but after my son’s marriage, my daughter-in-law cooks for me. When my son became old enough he got a job as an auto rickshaw driver, and has been supporting me ever since.” "

Among the widows whose husbands abused alcohol, although there may have been some psychological relief with their husband’s passing (particularly those who have been abandoned); they reported persistent suffering with “emotional pain” due to the great shame associated with their husbands’ death.

### Widowhood: challenges and opportunities for living healthy and fulfilling lives

#### Economic security

Economic security emerged as the key challenge. After losing their husbands, the women claimed that they had no desire to remarry. Only one woman remarried (and was subsequently widowed for a second time), she reported that her second marriage was problematic. The universal reason given for not remarrying was for the sake of their children, in Priya’s words: “I did not wish to remarry because my children are precious to me”. All the women in the sample had children; the age of widowhood ranged between 21 and 63 years of age with most women widowed during their late 20s or 30s (Table 1). Elsamma, for example, had 5 children by the time she was widowed at the age of 27 years. Without remarrying, the widows had to secure their economic well-being in other ways.

Three women had adult children – sons and daughters – who supported them; one of these women, Jamila a Muslim woman widowed at the age of 63, was also receiving her husband’s retirement pension (Table 2). Jamila, despite having some health problems, reported the least problems among the participants:

**Table 2:** Income generation activities and financial support among widows interviewed

**Pseudonym**	**Income generation activities**	**Government support**	**Community and family support**
Geeta	Self employed	-	10, 000 Rs. from Family welfare society, 750 Rs. from church, loan from SHG for buying milch cow
Elsamma	-	-	200 Rs. per year from children
Amina	Agriculture worker in coffee estate	-	-
Jamila	-	Husband’s retirement pension	Loan from kudumbrasree to buy chickens
Lata	*Kuli* worker	*	Loan from kudumbrasree to buy 10 chickens
Priya	-	-	Supported by son
Usha	Anganvadi assistant	Loan from housing board for house	Supported by daughters
Sumathi	Domestic worker	Widow pension	Supported by middle daughter
Leela	*Kuli* worker	Widow pension	-
Shantha	-	Widow pension	Supported by eldest daughter/son-in-law

Source: Qualitative survey.

* Has applied for widow pension, but it has yet to be sanctioned.

"“My husband had been a school teacher. I am getting his pension. My children are taking care of me. I am receiving treatment from Dr. Jayla at Salka Hospital. I take ordinary food. I receive everyone’s respect as an elder.” "

Without family support, the other respondents talked about their struggle to make ends meet. Three women were receiving the widow pension, which entailed Rs 110 per month, received in two lump sums each year (Table 2). Other respondents were not eligible, as a woman needs to meet three criteria: belonging to a household that is below the poverty line, have no living son aged 18 years or older, and has not remarried. The main drawback for these criteria is among women whose adult son does not care for the family. For example, Sumanthi discussed how her son does not provide any financial support for her and the family despite the many health problems faced by her and the family:

"“My son does not provide any financial support. A few days ago he visited us then left the household without assisting us even though I asked for money to pay for medicine for myself and his sister.”"

Sumanthi does collect the widow pension, which she said was facilitated by political connections as her husband had been working with the Communist Party of India (Marxist). But for other women, they complained about the difficulties faced in applying for the widow pension, such as travelling alone to the office and handling the administrative requirements. As Geeta recalled:

"“I had a bad experience with the Panchayat offices. I went for documents and the staff of the office asked me to come back in the evening and it was for bad intentions, I felt that they would harass me.” "

Shantha eventually received the widow pension, but only after a ward councillor intervened on her behalf:

"“I have been receiving the widow pension for the past three years. I applied a number of times for it but did not receive it. They say that they do not know the reason. Finally, my ward member applied for me and now I have it.” "

Six women work in low paying jobs as agricultural labourers (generally known as *kuli* workers who engage in wage labour), domestic workers, and child care providers (Table 2). At the time of her husband’s death, Usha was not able to meet the needs of her and her three children as an *anganvadi* assistant:

"“I received an honorarium of Rs. 50 per month, which was not sufficient to support my family. I took on additional work. I would work at the angavadi from 9:30 to 3:00, and in the evenings I would clean houses. On Saturdays and Sundays I would work as a kuli worker making 10–12 rupees per day.” "

Today, Usha’s life is easier because her children have grown up and are supporting her including her eldest daughter Beena. Leela had previously travelled to another district, finding work as a domestic help. She has since returned to live with her parents and is a *kuli* worker. We were also informed of the two women who had left the *panchayat* after becoming widows that one had gone to find work in a nearby district and the other had joined a convent.

One opportunity for economic security was to participate in a self help group, a form of microcredit program. Three of the respondents took loans from self help groups. These women talked about how these loans have helped them. In one case, Geeta had previously gone for wage work, but she was often taunted by men for travelling outside the house. After joining a *kudumbrasree* group (a self help group supported by the local government) she was able to purchase two milch cows with a loan:

"“I have been a member of Kudumbrasree for two years. I took a loan and bought a milch cow. I also have a second cow, both of which recently had calves. From the two cows I am able to extract 10 litres of milk a day. For each litre of milk I will receive about Rs. 10 selling to the Milk Society. I am happy with this, I am now self-sufficient.” "

Geeta further explained that her new job enables her to stay at home to care for her children. Jamila and Lata took loans to purchase chickens, which were used to supplement other sources of income. Other respondents, however, did not reap similar benefits from self help groups. Usha, for example, reported having to withdraw from a self help group due to time constraints:

"“Two years back I joined a self help group. I enjoyed being a member but I had to withdraw because of lack of time. I was working morning till evening six days a week. On Sundays I would clean and take care of my own home. I was also unable to participate in any of the activities outside of the meetings.”"

#### Social mobility and social isolation

The second key challenge was related to social mobility and social isolation. Among the younger respondents, they voiced their difficulty with new restrictions in their social mobility. This included attending certain religious and social activities, but also seeking health care, as illustrated by Geeta:

"“I am not allowed to go free and travel free. It is better to have a husband just for name sake at least. In this way others will not comment on me. The people are not allowing me to wear a decent dress in the absence of a husband. When my husband was alive I was taken care of very well and if I was ill, he took me for treatment at the hospitals…. Now I am not able to attend festivals and such activities because of the way people talk.” "

The older respondents talked about feelings of isolation. Elsamma, an elderly widow has five living children, yet she lives alone in a house provided by the government. She received few visitors and cooked for herself. Jamala lives with her son and his family, but still expressed feeling lonely. This situation improved for some women, however, after they joined a self help group. For Priya, becoming a member of a self help group opened up new social networks:

"“I joined RASTA [a local NGO] self help groups 7 years ago, replacing my daughter as a member in one group following her marriage. At that time I was quite lonely in the house and found self help groups gave me friendships.” "

For Priya, who did not take a loan (because she was not in need of a loan), it was also the social dimensions of the SHG that she felt was important for her. Leela has also continued as a member of a self help group because of the social aspects of the group, having no interest in taking a loan (because she was worried that she would not be able to repay the loan). Social networking and social support was cited by other women who had taken loans as an additional benefit to their membership.

#### Access to health care

The third key challenge voiced by the participants was related to health. The respondents talked about a range of health problems, the most common being respiratory problems, joint and body pain, and mobility difficulties. Despite facing health problems, most respondents revealed that they had difficulties meeting health care costs for themselves or for a family member. This is illustrated by Usha’s story:

"“I have some problems in my back and shoulders, bone disintegration, causing a great deal of pain. I think this is caused by pulling water from the well at the anganvadi. I visit the public hospital. The doctor told me that if I pay Rs. 2000 I could have a ring put in my back to help ease the pain. I cannot find the money for this. I also have uterus pain. At times I cannot urinate, I will then go for treatment from the doctor who will prescribe medicine. I will take this medicine until the urine comes back. After this, the pain and urinal dysfunctions will come back. The doctor has suggested she remove her uterus, for which I have not even questioned the cost.” "

Sumanthi needed to take care of her 35 year old daughter with a mental disorder requiring medicines costing Rs. 42 for five days. At the time of the interview, the condition of her daughter had worsened as the family did not have the money to purchase her medicine. Only Jamila, who had both her husband’s pension and the support of her children, was able to access the health care she needed.

## Discussion

### Widows in Kerala

Martha Chen, who has conducted pioneering research on widows in India, recalled her experience in Kerala where she was told by a well-known Communist leader that: “There is no such thing as widowhood in Kerala”. ([1] p. 180) The women in this study were not exposed to some of the more extreme forms of suffering and discrimination experienced in other regions of India, but their experiences illustrate their vulnerability to poverty, social exclusion, and ill health. In the following section, we develop a conceptual framework by synthesizing our findings with theoretical understandings of vulnerability and Amartya Sen’s entitlements approach. [18] We also draw on findings from research undertaken in other parts of India to promote a wider applicability of the framework.

### The conceptual framework

#### Vulnerability and the ‘shock’ of becoming a widow

Although vulnerability has been defined in various ways, it is generally meant to signify that there is a capacity for harm by an individual or group in response to a stimulus or risk. [19,20] Definitions have tended to vary across disciplines, diverging according to the focus on different aspects of risks. [21] We focus on vulnerability *to* poverty and ill health as a response to a ‘shock’, specifically the characteristics of a person or group in terms of “their capacity to anticipate, cope with, resist, and recover from” a shock [22], p. 269. To be vulnerable entails a sense of fragility, although one is still ‘intact’. [23] Fragility encompasses both the occurrence of a shock (or a stress) and the incapacity to cope with this shock, which we argue is attributed to insufficient entitlements.

Based on our findings in this study, we argue that becoming a widow appears to operate similarly to other ‘economic shocks’ or ‘health shocks’ in poor countries. In the case of a health shock, such as death of a working age household member or a chronic illness requiring long term care, households can face a double financial burden due to the loss of income of a breadwinner and the burden of health care expenses. [24,25] What is particular about widowhood is that financial burdens tend to fall entirely upon women. This is especially challenging in a society, such as India, where the employment opportunities for women are limited (and wage differentials are high; women are often paid half the wages of men) due to both the dynamics of the labour market and social norms. Health care expenses have been observed to be a major factor in the downward mobility of households. [26] Costs may operate as a barrier to accessing the necessary care, a situation found among some of the husbands who could not access appropriate care. Women who were married to men who abused alcohol faced economic hardships as their husbands’ diverted income from household needs to alcohol consumption. Even among those women with a husband, who abuses alcohol creates a situation in which they are essentially living as widows.

#### Stages of widowhood

The vulnerability of widows may be characterized into three phases. The initial phase occurs prior to her husband’s death. During this time, she may be confronted with caring for her husband (if he is ill) or dealing with his alcohol abuse. In both cases, women may need to assume financial responsibilities due to medical bills (in the first case) and the shedding of any financial responsibility from the husband (in the second case). Women may face other challenges during this stage. Women with husbands who abuse alcohol may also be victims of domestic violence. Although we did not explicitly explore this issue, as it is an extremely sensitive topic requiring special ethical and methodological considerations, [27] physical violence did arise as a consequence of alcohol abuse in the cases of Beena and Usha. Moreover, there is a growing literature demonstrating alcohol to be a determinant of domestic violence in India. [12,28] Additionally, while the prevalence of HIV/AIDS and other infectious diseases is relatively low in Kerala, in other parts of India, women may face the additional burdens of being infected themselves. [29] It is less clear how the loss of a husband due to an accident or non-stigmatising illness may influence the characterisation of this first phase, but will likely depend on the immediacy of these causes of death (e.g. a lengthy period of care giving in the case of a debilitating accident versus an accident that caused immediate death).

The second phase occurs when a woman’s husband dies. At this time, women must not only cope with the loss of their husband but also are faced with the challenges of ensuring the livelihood of their children, while coping with financial debts incurred from medical bills if the husband was ill for a long period of time or the social stigmatization if the husband committed suicide, or died from alcohol-related causes or socially stigmatizing diseases, such as HIV/AIDS. During this period, there appears to be little support beyond assistance from community, which the women in our sample felt was an important albeit temporary source of support, thereby increasing the vulnerability of widows and their children to impoverishment and poor health. Again, it is unclear from our study how the loss of husband from an accident or non-chronic illness may influence this phase; however, the sudden and unexpected loss of husband will likely exacerbate the intensity of the ‘shock’—both emotionally and financially—experienced by the woman and her family.

During the third phase, women may begin to learn to assume greater decision-making roles in their households. Enhancing women’s decision-making agency has been advocated as one approach to improving women’s status. [30] However, some researchers have cautioned that we should not assume that this should translate into women making individualised decisions; ‘joint decision-making’ when women make decisions in partnership with their husbands should also be considered a sign of women’s control. [31] While we did not explicitly study decision-making as part of this study, including not having information on women’s decision-making agency before and after becoming a widow, our results suggest that women found the initial period of having to assume all major household decisions to be stressful due to the competing demands that they faced. But over time, the women acquired more confidence in their decision-making skills, especially when they could integrate their children into the decision-making process, which suggests that individualised decision-making is not necessarily the ideal situation for all women. During the third phase, women were also taking on the role of the main breadwinner. In the absence of family support, widows enter the labour force or expand their activities, usually working long hours and often in less than ideal conditions. [1] Widows, particularly younger widows, face social stigmatisation and restrictions in their social and occupational mobility, limiting their employment opportunities as well as their participation in social and community activities.

#### Set of restrictions

As widows, women face a set of restrictions that increases their vulnerability. The *shame* of becoming a widow, which is compounded among certain causes of death (e.g. suicide; HIV/AIDS) where they may be blamed for their husbands’ death, can be debilitating for a woman, [2] Such shame can have a strong psychological impact, restricting her from appearing in public and engaging in social activities. For Geeta, taunts and the negative talk about her—being blamed for her husband’s suicide and being a widow—prevented her from attending social and cultural activities in addition to making it difficult for her to seek health care.

As a widow, a woman faces *social stigmatisation* and *social exclusion*. The level of stigmatisation may vary according to the social norms of the particular society, ranging from a ‘social death’ in certain parts of North India [1] to experiencing ridicule and taunts in Kerala, as discussed by the women in our study. Widows are restricted in their social mobility, confining her to her household. They may also be excluded from attending social and religious events.

*Restrictions in remarrying* are conditioned by social norms and can be linked to a woman’s caste and class status. In our sample, there was also a preference by women for not remarrying for the sake of their children; mothers would like to protect their children from possible neglect from a new husband who may not care for her children. [1] Since all of the women in our study had children their opportunities for remarriage were substantially lowered.

#### Responsibilities

As women lost their husband prematurely, they were faced with a heavy burden of responsibilities, which increased their vulnerability. This study identified numerous responsibilities. First, women were charged with the responsibility of *caring* for their chronically ill husband. (While in the Kerala context, chronic illness is the major cause of mortality, similar responsibilities for women would be found in societies that continue to face high levels of communicable disease.) Second, women who have had a husband with a lengthy illness are often faced with *paying off debts* incurred through medical bills. Third, women must often *make up for lost income*, due to the loss of the main breadwinner before the death of their husband. This may occur when a woman has a husband, either whose illness or injury prevented him from working, or who spends his money on alcohol instead of on household needs. Fourth, as widows, women (in the absence of an adult breadwinner) must take on the role of the *primary breadwinner*. This may mean either taking up employment or working additional jobs. Fifth, women are faced with becoming a *single parent*, often without additional family support. In our study, among the women who remained in their husbands’ village, there was no family support for the husband’s family in child rearing. Finally, in the absence of an adult son (or daughter) women are charged with assuming *household decision*-*making*, which can be very stressful, especially if they had not been part of household decision-making in the past, by either taking joint decisions with their husband or being responsible for certain household decisions (e.g. education of children).

#### Endowment and entitlement

Sen’s work is useful in helping to *explain* the vulnerability of widows. In the entitlement approach, Sen defines ‘endowment’ as encompassing the resources of a woman that condition her ‘entitlement exchange’, that is her command to exchange what she owns for a bundle of commodities, such as food or medicine. [18] Her entitlement set is the set of all potential combinations of goods and services that a woman can legally secure given her endowments. While the entitlement approach has been typically used for famine analysis, it is also a useful framework for understanding women’s rights in securing resources, both from her household and society, in order to increase her opportunities for health and well-being.

Widows tend to have a relatively small endowment (economic resources, social resources, social security), which increases their vulnerability by limiting a widow’s capacity to cope with the shock of becoming a widow. While the distribution of capital, land, and income will vary according to a woman’s caste and class, the restrictions placed on a widow may impede her claim on land and other resources. Strengthening *property rights* among women in India has been argued to be one of the key strategies to improving women’s status in general and widows in particular. [5,32] The lack of property rights has restricted women from securing their husband’s land following his death – often his family will reclaim the land. In general, widow’s inheritance of land from their husbands have been much higher in the south than in the north, although considerable diversity in widow’s claims have been found across northern states, which have been attributed to differences in public policy and ancient legal treatises. [5,33] In Kerala, property rights for women are among the strongest in the country. [32] In our sample, most of the women owned some land which was predominantly passed onto them from their husband (Table 2). However, this land was mostly house plots. Among the two women who had substantial land plots, both with forward caste affiliations, the land was not being used for any productive purposes. It is not clear whether women with larger landholdings did not use their land due to the quality of the land or limited capacities to earn income from their land.

*Employment restrictions* limit widows’ capacities to survive and to support their children. These limitations may arise, notably in the north, due to caste restrictions in working outside the household. [1] In Kerala, work opportunities are limited, especially for women, due to the labour market in which there are few employment opportunities outside the agricultural sector [3]. There is little job mobility to improve one’s job status and therefore extend her entitlement exchange. Employment opportunities in India are further restricted, especially among younger women, who face limited social mobility and threats of physical and sexual harassment in the workforce [1].

A social network, which may facilitate entitlement exchange, may be diminished upon widowhood as a woman faces greater restrictions on her social mobility and social stigmatisation. Social security in India for widows is predominantly through the widow pension, which can provide some relief. Although, there have been critiques that the pension is insufficient and fraught with bureaucratic challenges. Moreover, the presence of an adult son, even if he does not provide for his mother, will generally preclude a woman from claiming the widow pension.

In sum, widows face a high set of restrictions, high set of responsibilities, and limited endowment. This, in turn, constrains a widow’s capability to live a healthy and fulfilling life, [32] particularly during the early stage of widowhood. Evidence from this study, supported by other research, indicates that widows suffer from high levels of *material deprivation*, *limited job opportunities*, *social isolation*, *social exclusion*, *limited social mobility*, and *poor physical health*. [1] We also identified high levels of stress lowering her *mental peace*. This stress is not only linked to losing a spouse, which has been well documented in the literature on the health of widows in other contexts, [34] but also related to having to cope with the circumstance of the death of a husband (e.g. shame, blaming, debt) and the new responsibilities.

To be able to become and live a good life as a widow requires a capacity to cope with harsh and multiple challenges or *resiliency*. Three factors identified in our survey appear to promote resiliency. First, the support of adult children made a marked difference in the economic and social well-being of the women. While sons are generally viewed as the main source of support, we also found that daughters contributed to improving the lives of their mothers, which has been observed in other contexts. [31,35] Second, the widow pension was found to offer some relief, providing women with an independent source of income; also, confirmed by Dutta. [36] Third, self help groups provided economic and social opportunities for several women in the study. Self help groups, which are widespread in Kerala, may also help protect against exclusion from health care and the promotion of mental health [3].

#### Caste and class differences

Women who are wealthier and have higher caste affiliations are often at an advantage in society, tending to have higher levels of education and greater landholdings. However, in our study becoming a widow appears to have eclipsed differences across women. Irrespective of one’s caste or economic status, widowhood has a profound impact on a woman’s life raising many challenges. Caste norms can place restrictions upon forward caste women, limiting their opportunities to work or to take on certain economic activities. [1] This appears to be confirmed in our study. Higher levels of education may not necessarily be an advantage, especially in Kerala, where there are few job opportunities. The gap between education and opportunities to use this education has been found to have negative consequences for women, such as mental distress. Land ownership might not translate into a reality of utilising that land.

#### Limitations

This study included a small sample of women from a single *gram panchayat* in Kerala, which did not provide an opportunity to glean a portrait of widows across Kerala, given the internal diversity of the state. However, as this study builds on other research we have undertaken in the community, we have been able to provide an in-depth understanding of the realities of widows, who have been an understudied group in population health and human rights research. One of the benefits of building on our prior research was that we were able to use our census to select a sample based on a variety of socio-demographic characteristics—although since our selection was based on current marital status data, we were not able to identify any widow who has remarried. This lacuna is, however, negligible since once widowed, women tend not to remarry (which was confirmed by the women in our sample). Our sample did not include any woman whose husband died from a cause not associated with either alcohol or a chronic illness, such as an occupational or road accident or an infectious disease, which may have yielded different or modified profiles and requires further investigation.

## Conclusions

The women interviewed in this study face similar issues to other women in their community, such as exclusion from health care and restrictions in their social mobility, but their status as widows make them particularly vulnerable. Moreover, widows’ vulnerability—especially during the early stages of their widowhood— stems from an *accumulation* of challenges: limited resources, high levels of restrictions and responsibilities, and a lack of entitlements. Given that our research was undertaken in Kerala where women’s status is known to be relatively high and the provision of public services more equitably distributed than elsewhere in India, it suggests that advancing the agenda for improving health and rights of widows is of even greater importance at the national level. Our study supports policy suggestions that have been proposed by others working in India, notably recommendations stemming from workshops and conferences that brought together activists, scholars, lawyers, and widows themselves. [37] They advocated a multi-pronged approach that would simultaneously address material needs and social identity and respect. Family support is often insufficient to ensure an adequate living standard, there is a need to improve her (and her children’s) material opportunities by securing women’s property rights, expanding employment opportunities, and ensuring adequate social security benefits. Government interventions specific for widows have been sparse and the impact of available interventions inadequately analysed. [38] In our study, while some women who were eligible for the widow pension did receive it, the process to claim the pension was not always straightforward and tended to be facilitated in those cases where women had political connections. Programs should address short term health and welfare needs (i.e. the period immediately following the loss of their husband) of widows, as well as their longer term needs, since the majority of widows do not remarry and continue to face economic hardships and social stigmatization. The development of these programs should be done with the participation of widows (who are often excluded from policy dialogues) in order to ensure that they are appropriately tailored to their needs.

Improving widow’s social standing and ensuring they have their own identity will require another set of strategies, including public debates and campaigns, government initiatives that ensure that widows are protected from abuse and violence, and creating spaces for widows to organise and develop their own strategies for change. [1,39] In addition, international and domestic human rights treaties should be applied to address the various challenges that women face when their husband dies and to help protect the rights of widows [2].

We further propose a need for population level interventions that will address the underlying causes of the vulnerability of widows. The specific interventions may vary according to the particular health issues prevalent in a community (e.g. HIV/AIDS, chronic disease). Two key issues that emerged in the context of Kerala are inadequate social protection against health care costs and the negative consequences of male alcohol use. Community-based health insurance could help reduce the financial burden of health care of households, protecting against catastrophic health care costs and the incurring of debts that can impoverish households. Moreover, removing financial barriers to health care among poorer households can help to increase access to care, improving the chances and speed of recovery and the restoration of good health. These programs, however, need to be gender sensitive “as gender bias in treatment-seeking can persist even after the removal of economic barriers to access” [40], p. 552.

To address the causes and consequences of male alcohol abuse, community health promotion programs appropriate to the community context can be implemented. These programs could “tie-in” with successfully established self help group programs, helping to produce spill over effects. [41] Several pathways have been suggested linking women’s participation in microcredit and their health. [3] Implementing complementary interventions that address underlying causes of women’s ill health could enhance the effectiveness of these programs. The social solidarity of a woman’s organisation can help to address particularly sensitive issues, such as male alcohol use.

More research on widows in India and other countries will help to both elucidate the challenges faced by widows and encourage potential solutions. There is a need to undertake research in other contexts with diverse health challenges (e.g. HIV/AIDS), and socio-economic conditions (e.g. worsening economic deprivation), and cultural beliefs and practices. [2] The framework outlined in this paper could help serve to guide future research.

## Competing interests

The authors declare that they have no competing interests.

## Authors’ contributions

KM conceived and designed the study, led the field work and data analysis and wrote the first draft of the manuscript. SH and DN participated in data interpretation and the development of the theoretical framework and revised drafts of this manuscript. All authors read and approved the final manuscript.

## Pre-publication history

The pre-publication history for this paper can be accessed here:

http://www.biomedcentral.com/1472-698X/12/28/prepub

## Supplementary Material

Additional file 1 **Widow survey guide.** This additional file provides the guiding questions for the survey used in this study. Click here for file
