# Supplementary material for: Debt, shame, and survival: becoming and living as widows in rural Kerala, India
Source: BMC Int Health Hum Rights. 2012 Nov 6;12:28. doi: 10.1186/1472-698X-12-28 (PMC3517387; doi:10.1186/1472-698X-12-28)
Supplement: Additional file 1 — Widow survey guide. This additional file provides the guiding questions for the survey used in this study. [file 1472-698X-12-28-S1.doc]

**Widow Survey Guide**

*(Note to interviewers: these questions should be used as prompts for participants to share specific experiences and stories, as well as relating their experiences to norms of their community for questions 2-4).*

Guiding questions

1. What are the circumstances surrounding your widowhood?
   1. How did your husband die?
   2. When did this happen?
   3. How old were you at that time?
   4. Do you have children, how many? Do they live with you?
   5. What is your occupation?
   6. What is your caste/religion/tribe?
   7. What is your position in this household?
   8. Do you receive a widow pension? If yes, what is the payment schedule?
   9. Is there any dress code/social code of behaviour associated with widowhood in your community? Do you follow these rules?
2. Do you fee that you have any special health risks as a widow?
3. Has your life significant changed since becoming a widow (immediately following widowhood, and after some period of time), if so how?
   1. Health
   2. Access to health care and treatment
   3. Income/assets
   4. Living conditions
   5. Work
   6. Autonomy (mobility and decision-making)
   7. Food consumption
   8. Emotional well-being
   9. Social participation
4. How do you feel about support in your life, either financial or social?
   1. From family
   2. From government
   3. From community
